# Adolescent Riding Behavior During the COVID-19 Pandemic in Urban Area, Indonesia: A Cross-sectional Study

**DOI:** 10.4314/ejhs.v31i6.8

**Published:** 2021-11

**Authors:** Intan Zainafree, Suharyo Hadisaputro, Agus Suwandono, Bagoes Widjanarko

**Affiliations:** 1 Doctoral Program, Faculty of Public Health, Diponegoro University, Semarang, Indonesia; 2 Faculty of Medicine, Diponegoro University, Semarang, Indonesia; 3 Departement of Epidemiology, Faculty of Public Health, Diponegoro University, Semarang, Indonesia; 4 Departement of Health Promotion, Faculty of Public Health, Diponegoro University, Semarang, Indonesia

**Keywords:** Adolescent, Behavior, COVID-19, Road Traffic Accident, Road Safety Education, Indonesia

## Abstract

**Background:**

According to police reported crash, in 2020 there have been 510 road traffic accidents among adolescents aged 16–25 years. The problem is that although restrictions on social activities have been implemented, 9.80% of accidents have caused deaths in Semarang City. There were many factors that influence the occurrence of road traffic accidents; one of those is the poor knowledge about safe riding behavior. The aim of this study is to determine the factors that contribute to the occurrence of road traffic accidents in adolescents during the pandemic.

**Methods:**

This was a cross-sectional study, collected data using an online questionnaire distributed to adolescents aged 15–20 years in Semarang City, Indonesia. It was distributed during February-April 2021. The data included participant's demographic information, riding behavior, and knowledge about safe riding. We analyzed using chi-square and logistic regression to determine the most influential factors.

**Results:**

The sample included 725 participants with a mean age of 17.4 years (SD=0.97); 260 (35.9%) males. We have found that gender was associated with the incidence of road traffic accidents (AOR=1.455, 95% CI [1.048–2.020], P=0.025) after adjusting for experience road safety education, vehicle type, and knowledge of safe riding.

**Conclusion:**

It is necessary to carry out Road Safety Education efforts to male students during the pandemic to reduce the incidence of traffic accidents.

## Introduction

Indonesia is a developing country with a high population density; 144 people per km^2^ in 2019 (1). The rapid population growth in Indonesia is accompanied by an increasing number of vehicle ownership (2). This has an impact on higher mortality rates, one of which was due to Road Traffic Accidents (RTA) (3). The ratio between the number of vehicles and deaths due to RTA per 100,000 population has the same percentage; it is 200% (4). Adolescences are an age that is prone to RTA (5–10). In Indonesia, 4.9% of injuries due to traffic accidents occurred to adolescents (15–24 years) that which was 79.4% occurred to motorcyclists (11).

Central Java Province is one of the three provinces with the most vehicle ownership in Indonesia (12). During January-June 2020, RTA in Central Java reached 10,841 cases with caused in 1,726 deaths; it was the highest in Indonesia (13). The driving activities in the capital city of Central Java Province, Semarang City, should receive special attention. Because it is one of the four cities with the highest number of road traffic accidents in Central Java (13).

The incidence RTA at Semarang in 2017–2019 experienced an average increase of 15% and decreased by 18.4% in 2020 (14). It happened due to in 2020, the Semarang City Government had implemented restrictions of social activities *(Pembatasan Kegiatan Masyarakat)* to prevent the transmission of COVID-19 infection start from April 27 2020, until now (June 2021). In line with this, there were studies that have been carried out in several countries which show that restrictions on social activities have resulted in a 73% reduction in traffic violations and a 37% reduction in the incidence of traffic accidents (15). However, the number of traffic accidents for adolescent in 2020 at Semarang City only decreased by 6%; it is much lower than the previous year (13). Although, the restrictions of social activities in the field of education were still enforced through online teaching and learning systems *(Pembelajaran Jarak Jauh);* it does not necessarily reduce traffic accidents that occur in Semarang City.

During the restriction of social activities, traffic accidents experienced by students were 510 (54.3%) accidents of which 50 accidents were reported to have died. The Traffic Police of Semarang City *(Satlantas Polrestabes Semarang)* also reported that the type of vehicle involved in traffic accidents was mostly found, that is motorcycle with a total of 73% (16,17). This fact supports the results of a systematic review study that there has been a change driving behavior to become more risky during COVID-19 pandemic (18).

The various studies have shown that there were several risk factors of traffic accidents in adolescents. A research in Kazakhstan stated that the incidence of traffic accidents aged 0–19 years occurs mostly in the male driver (15). The current study found that adolescences were associated with risky behavior while riding a motorcycle, inexperience, high speed and no driving license increased the risk of motorcycle injury (19,20). The other studies mention a number of factors including the amount of exposure to driving, driving duration, social and peer factors, drugs, alcohol, and excessive speed often lead to traffic accident scenarios in adolescents (16,21). Meanwhile, the research in Indonesia found that having a driving license, driving attitude and behavior were factors that cause traffic accidents occurred in adolescents (17). The other studies have also found that motorcycle accidents occur due to unsafe adolescent behavior patterns, such as aggressive driving, neglect of traffic regulations, and lack of helmet use while riding (21,22)

Many previous studies have found the effect of limiting social activities on the incidence of RTA in several countries (18,23). However, there has been no research explained a specific age group of accidents of adolescent on safe riding behavior during the COVID-19 pandemic in urban areas. Therefore, this study aims to determine the factors that contribute to the occurrence of road traffic accidents in adolescents during the application of the online teaching and learning system due to the COVID-19 pandemic in urban areas, Indonesia.

## Methods and Materials

**Study design and setting**: This was a cross-sectional study, collected data using an online structured questionnaire during February to April 2021. The instrument in this study was prepared with reference to the motorcycle rider behavior questionnaire (MRBQ) and questions on the exam to get a driver's license with some modifications that were adapted to the population situation (26). This questionnaire has been tested for validity and reliability through limited study. The participants took an average of 10 minute answering the online questionnaire survey. The questionnaire consisted of three parts; the characteristics of participants, riding behavior and knowledge about safe riding.

The first part, characteristics of participants, consisted of the identity of participants, such as age, gender, household income and RTA experienced during the application of online teaching and learning systems due to the COVID-19 pandemic. The second part, riding behavior, consisted of twenty-five item questions, such as type of motorcycle, speed, experience get Road Safety Education (RSE), using handphone while riding, hitchhiking behavior and the driving license ownership. The last part of the questionnaire was the knowledge about safe riding, consisted of twenty item questions.

**Data collection**: Data collection was conducted by distributed online questionnaires to *whatsapp groups* of several schools in Semarang City (24,25). Inclusion criteria consisted of registered as a high school student; actively participate in the online teaching and learning system and currently living in Semarang. The results of the calculation of the minimum sample using the Slovin's formula (standard error of 5%) are 398 participants. After the time limit expired the number of questionnaires returned and filled out was 799 questionnaires. Finally, the number of completely filled out questionnaires is 725 and that will be included in the final analysis. We assumed 725 participants has been able to describe adolescents in Semarang City.

**Variables and measures**: Data collected from study participants included (a) sociodemographic information (age, gender, household income, RTA experienced during the application of online teaching and learning systems due to the COVID-19 pandemic); (b) reported the most frequent times of riding a motorcycle (06.00–11.59 am, 12.00–5.59 pm, 06.00–00.00 pm); (c) reported the vehicle type which is less or more than 500 cc; (d) reported the speed when riding a motorcycle, which is less or more than 60 km/hour; (e) ownership of driving license; (f) commit traffic violation; (g) safe riding behavior and (h) knowledge of safe riding.

The participants' riding behavior was measured using a scale of 1 to 3 which shows the answers always, sometimes, and never. The participants were said to have safe behavior if their answer meets a score above the average of the maximum total score. Twenty-item questions adapted from the MRBQ and consisting of a combination of true/false and multiple-choice answers were used to develop an index of safety riding knowledge scores. The participants were said to have high knowledge about safe riding if the total value obtained was above the average of all participant's answers.

**Statistical analysis**: The data are presented in frequency and percentage-based RTA experiences. *Chi-square* test was conducted to analyze the relationship between risk factors and the incidence of traffic accidents in adolescent. The *p* value <0.05 was considered statistically significant. The risk factors with *p* values lower than 0.05 were included in the backward stepwise method logistic regression analysis (likelihood ratio).

**Funding source and ethical consideration**: No funding was received in this study. Ethical clearance was obtained from Health Research Ethics Committee (KEPK) Universitas Negeri Semarang with code No. 078/KEPK/EC/2021. All the subjects involved have filled out an online informed consent and agreed to participate in it.

## Results

**Figure 1:**
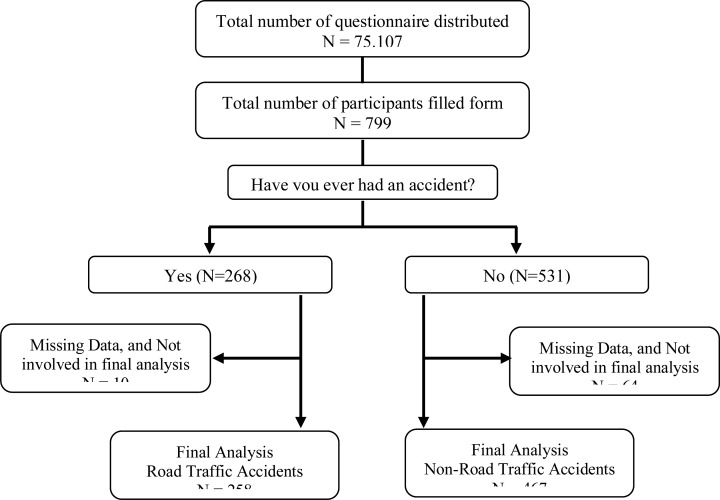
Participants' recruitment

Based on Figure 1, it showed that 799 participants there were 74 (9.2%) participant's answers that had missing data, because the questionnaire was not filled out completely by the participants, so they were not included in the analysis. Then the final sample included in the analysis was 725 participants. It was known that there are 258 (35.5%) participants who have experienced an accident and 467 (64.5%) of the participants has never had an accident during the restrictions of social activities.

Table 1 shows the characteristics of the participants. The average age of the participants was 17.4 (SD=0.97) and the majority were female 465 (64.1%). Most of the participants belong to the high-income group of 439 (60.6%) people.

**Table 1:** Characteristics of participants

Characteristics of Participants	Frequency (N=725)	Percent
**Age**, (yr, median)	17 (15 – 20)	-
**Gender**		
Male	260	35.9
Female	465	64.1
**Secondary Education Grade**		
Grade 10	155	21.4
Grade 11	221	30.5
Grade 12	349	48.1
**Household Income (minimum wage in Semarang, 2021)**		
High income (≥ IDR 2.810)	439	60.6
Low income (< IDR 2.810)	286	39.4
**Motorcycle Accident (N=258)**		
Fell of vehicle	81	31.4
Sideswipe collisions	73	28.3
Vehicle rollover	51	19.8
Head on collisions	37	14.3
Vehicle collides with another object	16	6.2

Table 2 shows factors of traffic accident in adolescent that the prevalence of have experienced road traffic accident was significantly higher in 8 items. Those were male students, never participated road safety education, the motorcycle used are 500–1000 cc, poor knowledge of safe riding, average speed of motorcycle riding is 50 km/hour, high household income, and risky riding behavior.

**Table 2:** Relationship between risk factor and road traffic accidents (RTA) among participants

	Experience of RTA						
				
Factors of RTA	Yes (N=258)		No (N=467)		*p-value*	PR	95%CI
	N	%	N	%			
**Gender**							
Male	79	30.4	181	69.6	0.029	0.697	0.505–0.964
Female	179	38.5	286	28.6			
**Secondary Education Grade**							
Grade 10	44	28.4	111	71.6	0.106	1	1
Grade 11	84	38.0	137	62.0		0.647	0.415–1.006
Grade 12	130	37.2	219	62.8		0.668	0.443–1.007
**Household Income**							
High income (≥ IDR 2.810)	171	39.0	268	61.0	0.019	1.459	1.063–2.003
Low income (< IDR 2.810)	87	30.4	199	69.6			
**Get a Road Safety Education**							
No	67	29.6	159	70.4	0.025	1.472	1.050–2.063
Yes	191	38.3	308	61.7			
**Riding Frequency each week**							
Daily	158	38.5	252	61.5	0.165	1	1
2–3 times each week	36	31.3	79	68.7		1.376	0.885–2.139
less than once a week	64	32.0	136	68.0		1.332	0.932–1905
**Most frequent riding locale**							
Outside city	48	18.6	85	18.2	0.945	1	1
Inside city	73	28.3	128	27.4		0.990	0.628–1.562
neighborhood	137	53.1	254	54.4		1.047	0.695–1.340
**Time of Mostly Frequent**							
06.00 – 11.59 a.m	48	30.8	108	69.2	0.201	1	1
12.00 – 17.59 p.m	148	35.6	268	64.4		0.805	0.542–1.194
06.00 – 00.00 p.m	62	40.5	91	59.5		0.652	0.408–1.042
**Vehicle type (cc)**							
500–1000	46	46.9	52	53.1	0.012	1.732	1.127–2.662
100–250	212	33.8	415	66.2			
**Average Speed (km/h)**							
≥ 50	81	42.4	110	57.6	0.022	1.485	1.058–2.084
< 50	117	33.1	357	66.9			
**Commit traffic violation**							
Yes	47	37.3	79	62.7	0.658	1.094	0.735–1629
No	211	35.2	388	64.8			
**Early age started riding (years)**							
< 17	242	35.6	438	64.4	0.996	1.001	0.533–1.881
≥ 17	16	35.6	29	64.4			
**Safe Riding Behavior**							
Unsafe	136	40.7	198	59.3	0.008	1.514	1.116–2.056
Safe	122	31.2	269	68.8			
**Driving License**							
No	175	34.4	339	66.0	0.117	0.796	0.572–1.108
Yes	83	39.3	128	60.7			
**Level Knowledge of Safe Riding**							
Poor	104	44.3	131	55.7	0.001	1.732	1.257–2.386
High	154	31.4	336	68.6			

Table 3 shows the results of the logistic regression analysis that gender was associated with the incidence of traffic accidents (AOR=1.455, 95% CI [1.048–2.020], P=0.025) after adjusting for experience road safety education, vehicle type, and level knowledge of safe riding.

**Table 3:** The effects of risk factor on road traffic accidents among participants

Factors of RTA	*B*	AOR {95%CI)	*p-value*
**Vehicle type (cc)**	-0.564	0.569 (0.368–0.879)	0.011
**Level Knowledge of Safe Riding**	-0.544	0.580 (0.420–0.801)	0.001
**Gender**	0.375	1.455 (1.048–2.020)	0.025

**Notes**: Hosmer and Lemeshow test: χ2= 0.685, P = 0.877, Nagelkerke R^2^ = 0.042

95% CI, 95% confidence interval; AOR, adjusted odds ratio

## Discussion

Semarang is the one of the urban areas in Central Java Province with a high incidence of traffic accidents. That was because Semarang has a high population and ownership of transportation (28). It is contrast with research conducted in European countries that urban areas have a lower accident rate than rural areas (27). We found that 35.5% of adolescents experienced traffic accidents during the pandemic such as vehicle falls, side collisions, vehicle rolls, head collisions and vehicles colliding with other objects. Statistically, gender was the most associated risk factor. Our findings show that males have a 1.502 times risk of having a traffic accident than females. The another study reported a high male-to-female ratio in motorcycle accident victims [28:1 and 16.9:1] (29,30). The another research report states that the reason men have a higher risk of having a traffic accident than female was the tendency of male to take risky behaviors more often (31), using a type of motorcycle with a high engine (> 550cc) (31), young male have higher impulsiveness and 2 decision-making styles (32) and aggressive driving behavior compared to young female (33).

We found that adolescent's participation in road safety education has an effect on traffic accidents. Statistically, based on table 2 participants who never got road safety education had a 1.472 times greater risk than those who had got road safety education. It was in line with previous research which found that with educational approaches to the driver, relatively small but still statistically significant reduction in road accidents (34). However, the efficiency and positive effect of RSE on overall road safety is still unknown (35).

The other findings in this study that most of the participants who used 500–1000cc motorcycles and unsafe riding behaviors had experienced of RTA. The current study adds to the evidence and reinforces previous research which concluded that average driving speed and bad driving safety behavior while driving are the human eror factors in the incidence of RTA's (7,8,27,36). In line with this study, adolescents who ride 500–1000cc motorcycles with an average driving speed of 60 km/hour; this is because adolescents have higher aggressive driving behavior (33).

The Research in Missouri (18) shows no reduction in RTA's resulting in serious or fatal injuries during a COVID-19 pandemic, a potential reason is increased traffic speeds due to lower congestion (16,37). Increasing traffic speeds can increase the number of serious or fatal road traffic accidents thus negate the effects of reduced traffic (18). There are probably other contributing factors such as a greater number of drivers under the influence of alcohol and drugs, economic pressure on drivers to save time, changing in road-safety publicity campaigns, police levels, the amount of fines, and an increase in traffic speed of heavy vehicles without volume changes (37,38).

The risky riding behavior in this study was the driver doing other activities such as using a handphone. That devices which are currently the main human needs, make it impossible for a person to easily let their hands off, as well as participants who often operate handphones anywhere and anytime (6,39). We found that doing other activities such as operating handphones, smoking and excessive interaction with drivers is at risk of experiencing 1.514 traffic accidents. This is because operating a handphone and smoking interferes with the driver's concentration and reflexes while riding. The results of the study found that some participants operate their handphones while riding, such as seeing at the same time using live maps applications is 67.8%, making calls and answering phone calls 23.3%, listening to music using earphones 21.6%, sending and replying to chats 20.8%, taking videos 14.5% and making live status and live streaming social media 11% (39).

Another activity that was carried out while riding in this study was smoking. Our survey results showed 6% of participants smoked while riding a motorcycle. In addition, other activities were also found that could affect the concentration of the driver, such as talking with the driver 80.9%, joking with the driver 7.8% and 1.1% of participants taking photos or videos with the driver. This finding is in line with research conducted by National Highway Traffic Safety Administration (NHTSA) that 7% of deaths in accidents due to distraction while driving are adolescents aged 15–19 years (40), on other findings, it is more specific that driving distraction experienced by drivers. Adolescents can cause collisions, especially in female drivers (16,36).

Another factor that we found is that the level of household income has an effect on the incidence of RTA. Statistically, participants with high household income have a risk of 0.307 times greater than those who have low household income. This is different from previous research that higher incomes have fewer traffic accidents (41).

It is necessary to carry out road safety education efforts to male students during the pandemic to reduce the incidence of traffic accidents. The strength of this research was to provide information on adolescents riding behavior during the COVID-19 pandemic. The limitation of this study is not providing information related to death and severity due to traffic accidents in adolescent during a COVID-19 pandemic. Hence, it was necessary to conduct similar research by including other factors such as environmental factors, vehicles, and road conditions.
